# Genetic analysis assists diagnosis of clinical systemic disease in children with excessive hyperopia

**DOI:** 10.1186/s12887-021-02992-7

**Published:** 2022-05-24

**Authors:** Shijin Wen, Xiaoshan Min, Ying Zhu, Xia Zhou

**Affiliations:** Eye Center of Xiangya Hospital, Hunan Key Laboratory of Ophthalmology, Central South University, Changsha, Hunan Province China

**Keywords:** Genetic analysis, SLSN5, IQCB1, MCOP6, PRSS56, Exotropia, Excessive hyperopia

## Abstract

**Background:**

A thorough examination (especially those including visual functional evaluation) is very important in children’s eye-development during clinical practice, when they encountered with unusual excessive hyperopia especially accompanied with other possible complications. Genetic testing would be beneficial for early differential diagnosis as blood sampling is more convenient than all other structural imaging capture tests or functional tests which need children to cooperate well. Thus genetic testing helps us to filter other possible multi-systemic diseases in children patients with eye disorder.

**Case presentation:**

A 3-year-old and an 8-year-old boy, both Chinese children clinically manifested as bilateral excessive hyperopia (≥+10.00), severe amblyopia and exotropia, have been genetically diagnosed as Senior-Loken syndrome-5 (SLSN5) and isolated posterior microphthalmos (MCOP6), respectively.

**Conclusions:**

This report demonstrates the importance of genetic diagnosis before a clinical consult. When children are too young to cooperate with examinations, genetic testing is valuable for predicting other systemic diseases and eye-related development and for implementing early interventions for the disease.

## Background

High hyperopia, defined as a type of refractive error of more than or equal to + 5.00 diopters (D), is usually accompanied by microphthalmia (axial length, AL < 21 mm) [1–3]. Microphthalmia can be divided into two categories: nanophthalmos, characterized by a short axial length accompanied by a proportionally shortened anterior segment, a small cornea, a shallow anterior chamber and a narrow anterior chamber angle; and posterior microphthalmos, which is characterized by a short axial length accompanied by a normal-sized anterior segment and a normal anterior chamber depth, along with a thickened sclera and choroid on ultrasound examination [2]. The normal corneal diameter is used to differentiate posterior microphthalmos from nanophthalmos with a microcornea [4]. High hyperopia is often accompanied by low vision, amblyopia, strabismus, and angle-closure glaucoma [5–7].

Based on population-based research, the prevalence of high hyperopia (≥ + 4.00 D) in children from 6 months to 6 years of age is 3.2% [8]. Excessive hyperopia (≥ + 10.00 D) is rarely reported. It is currently believed that genetic factors play an important role in the development of high hyperopia [9–12], and other hereditary eye diseases, such as microphthalmia [13, 14], Senior-Loken syndrome-5 (*SLSN5*) [15, 16], Leber congenital amaurosis [17] and retinal dystrophy [18, 19], can also manifest as high hyperopia.

Here, we report two cases of hereditary eye disease with binocular excessive hyperopia, severe amblyopia, and exotropia as prominent clinical manifestations that were confirmed by genetic diagnosis as *SLSN5* and isolated microphthalmia 6 (MCOP6). These diagnoses were used to predict eye and body development.

## Case presentation

The clinical characteristics and exome sequencing results of the two patients are summarized in Table 1.

**Table 1:** Clinical characteristics and exome sequencing results of the two patients

Information	Case 1	Case 2
Age	3 yr	8 yr
Sex	Male	Male
BCVA (OD/OS)	HM 50 cm/HM 50 cm	0.1+/0.1
Hyperopia	+ 10.00 D (OU)	+ 14.50 D (OU)
Amblyopia	Severe	Severe
Exotropia	-30°	-5° ~ −20°
Nystagmus	Yes	No
AL (OD/OS)	18.5 mm/18.3 mm	15.9 mm/15.9 mm
ACD (OD/OS)	2.7 mm/2.7 mm	2.9 mm/2.9 mm
LT (OD/OS)	3.5 mm/3.6 mm	4.2 mm/4.3 mm
Keratic curvature (OD/OS)	/	48.4 D/48.0 D
Fundus	The edge of the optic disc and its surroundings seemed to be thickened and the reflection of the macula was absent	Crowded optic disc, small optic cup and narrowed blood vessels around the macula were present
Mutated genes	IQCB1	PRSS56
Pathogenic variants	c.1090C > T, p.(R364*) and c.1333C > T, p.(R445*) complex heterozygous mutations	*c.632G > C, p.(C211S)* and *c.1066dup, p.(Q356Pfs)* complex heterozygous mutations
Disease(s)	SLSN5(OMIM# 609254)	MCOP6(OMIM#613517)

*BCVA* Best Corrected Visual Acuity, *AL* Axial Length, *ACD* Anterior Chamber Depth, *LT* Lens Thickness

Case 1, a 3-year-old boy from China, was born with poor vision that prevented him from tracking moving objects in front of his eyes. He had binocular excessive hyperopia (+ 10.00 D), constant exotropia (− 30°), loss of close fixation and positioning, horizontal nystagmus and an insensitive light reflex. The optic disc and its surroundings seemed to be thickened, and reflection in the macula was absent. No obvious abnormality existed in the rest of the eye structure.

**Fig. 1:**
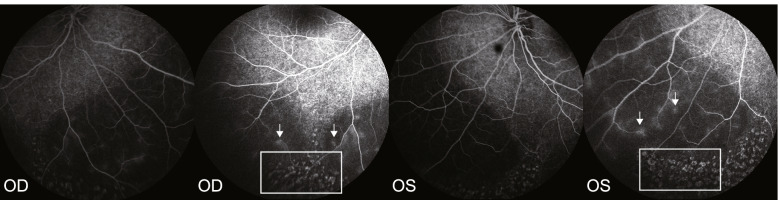
FFA (case 1) shows a large number of spot-like transmitted fluorescent foci and pigment-shaded fluorescent foci in the retina at the venous stage in both eyes, the peripheral retinal blood vessels were slightly thin, and several transmitted fluorescent foci were mixed with pigment-shaded fluorescent foci in the peripheral retina (rectangle), while light fluorescence leakage could be seen in the local blood vessels of the peripheral retina in the late stage (arrow)

**Fig. 2:**
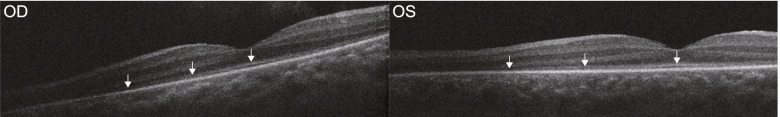
OCT (case 1) showed that the outer retinal band (including the ellipsoid zone band) was absent far from the fovea of the macula (arrow indicates the outer retinal band)

His visual evoked potential (VEP) indicated that the P2 peaks were delayed under all white, red, and blue photopic stimuli. Fundus fluorescein angiography (FFA) results are shown in Fig. 1. Optical coherence tomography (OCT) results are shown in Fig. 2. The patient was born at 39 weeks gestation with a birth weight of 3.35 kg. No toxic exposure, consanguineous marriage or a related family genetic history was reported.

**Fig. 3:**
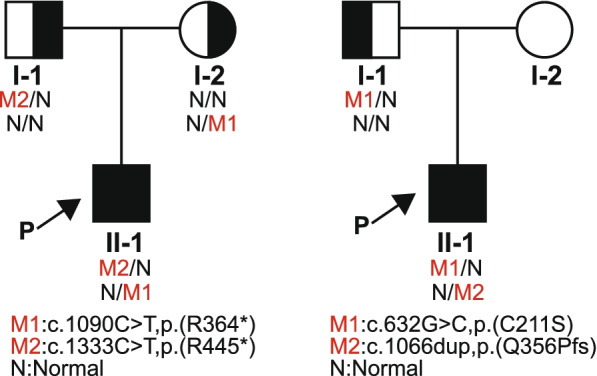
The first figure shows the pedigree and IQCB1 mutations of case 1. I-1: the father, I-2: the mother, II-1: the case 1. M1: c.1090C > T, p.(R364*), M2: c.1333C > T, p.(R445*), N: Normal. The second figure shows the pedigree and PRSS56 mutations of case 2. I-1: the father, I-2: the mother (she did not previously undergo genetic testing), II-1: case 2. M1: *c.632G > C, p.(C211S)*, M2: *c.1066dup, p.(Q356Pfs)*, N: Normal

Exome sequencing results revealed the autosomal recessive disease *SLSN5* (OMIM# 609254) caused by an IQCB1 mutation. There were c.1090C > T, p.(R364*) and c.1333C > T, p.(R445*) complex heterozygous mutations in the IQCB1 gene, both of which are nonsense mutations; the former was inherited from the mother, and the latter was inherited from the father (Fig. 3). c.1090C > T, p.(R364*) is included in the Human Gene Mutation Database (HGMD) and ClinVar database and is classified as pathogenic and has been reported in many studies related to *SLSN5* [16, 20–22]. c.1333C > T, p.(R445*) is a novel variant not reported in the gnomAD database; however, the HGMD and the ClinVar database include multiple nonsense mutations or frameshift mutations after the termination position of this mutation, and it is classified as a pathogenic/probable pathogenic mutation. In this case, according to the American College of Medical Genetics and Genomics (ACMG) standards (data from VarSome) [15, 23], both c.1090C > T, p.(R364*) and c.1333C > T, p.(R445*) are pathogenic variants of *SLSN5*.

Case 2, an 8-year-old boy from China, was diagnosed with enophthalmos at the age of 6 months. He had binocular excessive hyperopia (+ 14.50 D), microphthalmia with a reduced anterior segment, and intermittent exotropia with a variable deviation (− 5° ~ − 20°). A crowded optic disc, small optic cup and narrowed blood vessels around the macula were present. No obvious abnormality existed in the rest of the eye structure. He had no disability in outdoor exercises and activities.

**Fig. 4:**
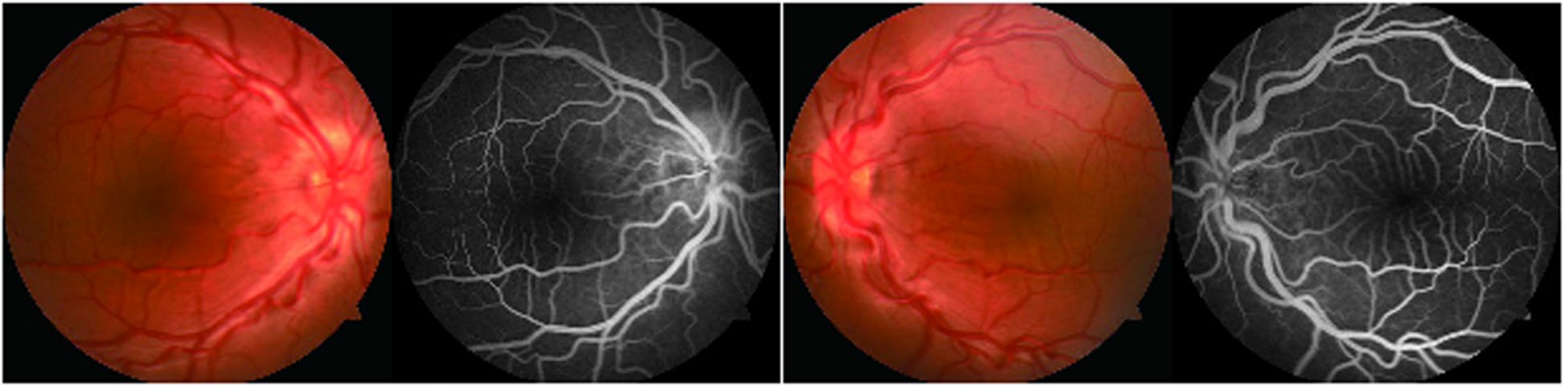
Fundoscopy and FFA (case 2) showing crowded optical discs, unclear boundaries of the optic papilla, tortuous vessels, and an abnormal foveal avascular zone. The fluorescence distribution was uneven, but there was no obvious fluorescence leakage

Fundoscopy and FFA results are shown in Fig. 4. The patient was born at 37 weeks gestation with a birthweight of 3.15 kg. No toxic exposure, consanguineous marriage or a relevant family genetic history was reported. Exome sequencing results revealed an autosomal recessive disease, MCOP6 (OMIM# 613517), caused by a PRSS56 mutation. There were *c.632G > C, p.(C211S)* and *c.1066dup, p.(Q356Pfs)* complex heterozygous mutations in the PRSS56 gene. The former is a missense mutation inherited from the father, and the latter is a frameshift de novo mutation (Fig. 3). *c.632G > C, p.(C211S)* is a very rare variant (gnomAD minor allele frequency (MAF) 0.00006, ID: rs1361878483) and is not present in the ClinVar database. *c.1066dup, p.(Q356Pfs)* is included in the HGMD and ClinVar database and is classified as pathogenic and has been reported in many studies as being related to microphthalmos. According to the ACMG standards (data from VarSome) [24, 25], *c.632G > C, p.(C211S)* and *c.1066dup, p.(Q356Pfs)* are pathogenic variants of MCOP6 [26–28].

## Discussion and conclusion

Whole-exome sequencing (WES) uses high-throughput technology to sequence genomic DNA extracted from samples of subjects, simultaneously analyzes the coding regions of more than 20,000 genes that include almost every known coding exon in the human genome, and then references them to the reference sequence of the human genome to identify variants in individual DNA that may be related to a disease [29, 30]. At present, WES has allowed great achievements in screening for carriers of recessive genetic diseases, the risk assessment of hereditary tumors, and screening for high-risk diseases described in the ACMG standards. This study used WES to diagnose two rare autosomal recessive diseases in Chinese patients.

The most common clinical manifestations of IQCB1 mutation-related *SLSN5* include early-onset retinal pigment degeneration, Leber congenital amaurosis and juvenile nephronophthisis. Early-onset retinal pigment degeneration mainly manifests as nystagmus or blindness at birth or within 2 years after birth [31, 32]. Because the IQCB1 mutation affects the development of photoreceptor cells, it will seriously impede the development of visual function in the first year of life and lead to severe visual impairment.

Ronquillo et al. [28] demonstrated that rod cells were completely degenerated at 30 days postpartum, cone cells began to degenerate at 6 days postpartum, and only cone cells remained after 30 days postpartum in mice in which the IQCB1 gene was knocked out. An electroretinogram (ERG) revealed a large number of lost cone and rod cells (mainly rod cells), pigment migration to the outer nuclear layer, and no blood vessels. Photophobia, nystagmus and hyperopia appear in the first few years of life or in late childhood [16, 31, 32]. Retinal pigmentation is often detected at the age of 3–8 years (it is probable that children in this age group start to have clear complaints), manifesting mainly as night blindness. As the disease progresses, only central visual acuity is maintained, and only a few cone cells can maintain their function. Therefore, it is inevitable that peripheral vision diminishes in the late stages and that a complete loss of vision occurs in the final stage [26].

Juvenile nephronophthisis manifests mainly as an end-stage renal disease before the age of 20, at a median age of 13 years. Polyuria, thirst, and nocturia may occur at the age of 6, while the urine analysis of most children is normal except for a decline in osmotic pressure. Anemia and growth stagnation might develop later. At an average age of 9 years, serum creatinine slightly increases, and the final stage of renal disease occurs within a few years. Typical ultrasound of the kidney shows a normal or reduced kidney size, enhanced echogenicity, and the formation of cortical medullary cysts in the late stage of the disease [27, 33, 34].

In case 1 reported here, the parents found that the boy was unable to track moving objects, with severely low vision at 6 months; then, he gradually developed nystagmus, and his best corrected visual acuity (BCVA) was HM 50 cm (OU). Finally, binocular high hyperopia was detected. FFA (Fig. 1) showed a large number of spot-like transmitted fluorescent foci and pigment-shaded fluorescent foci in the retina at the venous stage in both eyes. The peripheral retinal blood vessels were slightly thin, many transmitted fluorescent foci were mixed with pigment-shaded fluorescent foci in the peripheral retina, and light fluorescence leakage could be seen in the local blood vessels of the peripheral retina in the late stage, providing evidence of binocular retinitis pigmentosa and supporting the genetic diagnosis.

OCT showed that the outer retinal band (including the ellipsoid zone band) was absent far from the fovea of the macula [35]. After half a year of basic amblyopia training, his visual acuity did not significantly improve. His basic clinical manifestations were consistent with those in the literature. In addition, cranial magnetic resonance imaging (MRI) showed mild ischemia of white matter around the posterior horns of the bilateral ventricle and Cisterna magna. According to the abdominal color Doppler ultrasound, the internal diameters of both renal arteries were 4.0 mm, the peak systolic velocities (PSVs) were 52 cm/s (right) and 54 cm/s (left), the end diastolic velocities (EDVs) were 23 cm/s (right) and 17 cm/s (left), the resistance indices (RIs) were 0.56 (right) and 0.67 (left), the kidney sizes were 86 × 36 mm (right) and 90 × 41 mm (left), and there was no cysts or lumps in either kidney and no obvious abnormality in vascular or renal morphology. Ultrasound bone sonometry indicated that the sound of speed (SOS) in the middle tibia was 3545 m/sec. No clinical manifestations associated with juvenile nephronophthisis were found on clinical laboratory or imaging examinations.

Researchers [36] found that all 11 patients diagnosed with Leber congenital amaurosis had high hyperopia, 7 of whom had renal dysfunction and were diagnosed with Senior-Loken syndrome. It is estimated that end-stage renal disease caused by the IQCB1 mutation is inevitable, and the range of ages at which end-stage renal disease commences should be extended to between ages 3 and 50. The boy described herein is still young (3 years old), and long-term follow-up related to his kidney function and morphology is necessary, although there are currently no positive signs.

MCOP6 manifests clinically as high hyperopia and a short axial length and is commonly accompanied by mildly to moderately reduced visual acuity and anisometropic or strabismic amblyopia [1]. In addition, an almost normal-sized anterior structure of the eye with steep corneal curvatures, a shallow anterior chamber, thick lenses, and a thickened scleral wall are observed [1, 37]. Because the position of the eyes is relatively deeper than that of normal eyes, the palpebral fissures appear relatively narrow. The fundus presents crowded optical discs, tortuous vessels, and an abnormal foveal avascular zone [38]. In addition, papillomacular folds are reported frequently [39–42]. In this case, the boy was diagnosed with enophthalmos at 6 months, but the diagnosis was not taken seriously. At the age of 8 years, he was found to have a short axial length (OD 15.9 mm/OS 15.9 mm), a normal white to white (WTW) distance (OD 11.9 mm/OS 12.0 mm), binocular excessive hyperopia (+ 14.50 D), steep corneal curvatures (48.4 D/48.0 D), a shallow anterior chamber (2.9 mm/2.9 mm), and thick lenses (4.2 mm/4.3 mm), accompanied by severe amblyopia and intermittent exotropia. Crowded optical discs, unclear boundaries of the optic papilla, tortuous vessels, an abnormal foveal avascular zone, uneven fluorescence distribution and no obvious fluorescence leakage were also present on fundoscopy and FFA (Fig. 4). These clinical manifestations are consistent with those in the literature. Basic amblyopia training was performed. In addition, the binocular pressure of the patient was 15 mmHg (OD) and 14 mmHg (OS) at the time of consultation. Although the results were within the normal range, long-term monitoring is still required. Ultrasound biomicroscopy (UBM) showed a wide temporal anterior chamber angle (the angle of the other anterior chamber was unclear due to poor cooperation of the child).

PRSS56 was identified by Gal et al. in 2011 [13] as the pathogenic gene of MCOP6. To date, its molecular biological pathogenic mechanism remains unclear. Nair et al. [43] agreed that matrix metalloproteinases, as endopeptidases, can contribute to growth, development and wound healing by directly or indirectly affecting extracellular matrix (ECM) processing and degradation [44]. Trypsin-like serine protease, encoded by PRSS56, may affect eyeball size by affecting the ECM, and Gal et al. [13] proposed that the proteolytic activity provided by the protein encoded by PRSS56 is essential for the development of the vitreous and the sclera and may be related to the pathogenic mechanism.

In addition, PRSS56 mutations are closely related to angle-closure glaucoma [25, 43, 45] by affecting the structures of the trabecular meshwork and iris. The intraocular pressure in case 2 is still normal, but routine monitoring of his intraocular pressure, as well as his anterior chamber angle structure, is necessary during his development. At present, a targeted amblyopia treatment based on refractive correction is being administered, as we should pay close attention to improving his poor visual function due to the developmental impact of microphthalmia. The biological measurements of his eyeballs need to be performed regularly to detect other complications as early as possible and to take appropriate steps to reduce their negative influences.

In summary, these two cases that similarly manifested as excessive hyperopia were diagnosed as hereditary eye diseases, further supporting the significant value of genetic diagnosis in the management of rare pediatric ophthalmic developmental diseases. It is difficult to perform FFA, ERG and other invasive examinations of the eyes and the whole body on young children with poor vision (the second child experienced nausea and vomiting when FFA was conducted, and the FFA images were incomplete). Relatively speaking, genetic diagnosis is simple, and it is very beneficial for accurately diagnosing diseases with complex and diverse clinical manifestations and for clarifying the direction of treatment. The first child was found to have retinitis pigmentosa after the genetic diagnosis of *SLSN5*, which is very meaningful for his clinical diagnosis and early intervention. At the same time, genetic diagnosis can also help doctors better evaluate the prognosis of the disease and provide evidence for whether active intervention is needed. Pediatric ophthalmologists should consider the possibility of genetic diseases when faced with a series of symptoms and perform related genetic tests to achieve a correct diagnosis in a timely fashion. This could lead to an early diagnosis of the underlying systemic disease and help achieve better developmental conditions for the child. A comprehensive understanding of the whole system and eye diseases is crucial for pediatric ophthalmologists when treating ocular conditions such as a superhigh degree of hyperopia, strabismus and very severe amblyopia.

## Data Availability

The datasets used and analyzed during the current study are available from the corresponding author on reasonable request.

## Abbreviations

*SLSN5*: Senior-Loken syndrome-5
MCOP6: Isolated posterior microphthalmos
FFA: Fundus fluorescein angiography
OCT: Optical coherence tomography
HGMD: Human Gene Mutation Database
WES: Whole-exome sequencing
ERG: Electroretinogram
BCVA: Best corrected visual acuity
AL: Axial length
ACD: Anterior chamber depth
LT: Lens thickness
MRI: Magnetic resonance imaging
PSV: Peak systolic velocity
EDV: End diastolic velocity
RI: Resistance index
SOS: Sound of speed
WTW: White to white
UBM: Ultrasound biomicroscope
ECM: Extracellular matrix
